# Psychometric Assessment of Screening Measures for Depression, Anxiety, Somatization, and Life Satisfaction in Honduran University Students

**DOI:** 10.3390/bs15111546

**Published:** 2025-11-13

**Authors:** Miguel Landa-Blanco, Raquel Mejía-Sánchez, Yarani Echenique, Dilcia Reyes-Murillo, Lina Mendoza-Recarte, Carolina Neves, Eliana Fuentes-Mendoza, Marcio Alexander Castillo-Díaz

**Affiliations:** School of Psychological Sciences, Faculty of Social Sciences, National Autonomous University of Honduras, Tegucigalpa 11101, Honduras; ramejias@unah.hn (R.M.-S.); yarani.echenique@unah.edu.hn (Y.E.); dilciareyesm96@gmail.com (D.R.-M.); lina.mendoza@unah.edu.hn (L.M.-R.); carolina.barrantes@unah.edu.hn (C.N.); eliana.fuentes@unah.edu.hn (E.F.-M.); marcio.castillo@unah.edu.hn (M.A.C.-D.)

**Keywords:** anxiety, depression, somatization, satisfaction with life, mental health screening, university students, Honduras

## Abstract

This study evaluated the structural, convergent, and discriminant validity, invariance, and internal consistency of the Generalized Anxiety Disorder—7 (GAD-7), Patient Health Questionnaire—9 (PHQ-9), Somatic Symptom Scale—8 (SSS-8), and Satisfaction with Life Scale (SWLS) among 910 students at the National Autonomous University of Honduras. Mental health issues are common among university students, with anxiety, depression, and somatization often co-occurring from a transdiagnostic perspective. Life satisfaction, meanwhile, is recognized as a protective factor for mental well-being. In response to rising psychological distress among university students, reliable mental health screening tools are critical for early detection and intervention. Confirmatory factor analysis (CFA) supported the unidimensional structure of each scale, while multigroup CFA demonstrated gender invariance. Women reported higher anxiety, depression, and somatization scores, whereas men had higher life satisfaction. Internal consistency, measured by McDonald’s Omega (*Ω*) and composite reliability, was excellent for GAD-7 (*Ω* = 0.927), PHQ-9 (*Ω* = 0.919), and SSS-8 (*Ω* = 0.873). Convergent and discriminant validity were supported through significant correlations: GAD-7, PHQ-9, and SSS-8 were positively correlated with each other, and negatively correlated with SWLS. These findings confirm that the four scales are psychometrically sound instruments for evaluating mental health in Honduran university students.

## 1. Introduction

Mental health issues are highly prevalent across the Americas, presenting significant challenges for individuals and societies (WHO, 2022). Scholars conceptualize health as more than just the absence of disease; it encompasses a holistic sense of well-being that integrates physical, social, and mental dimensions (Larsen, 2022; Törrönen, 2025; Vera Villarroel et al., 2012). In this sense, well-being is a multidimensional construct that encompasses individuals’ overall psychological, emotional, and social functioning, including their sense of purpose, life satisfaction, and capacity to manage stress and maintain fulfilling relationships. It reflects not only the absence of distress but also the presence of positive emotions, personal growth, and alignment with meaningful goals and values (Al-Hendawi et al., 2024). This position frames what is known as dual-factor models of mental health, which propose that mental health is best understood through the simultaneous consideration of both negative indicators, such as psychopathology, and positive indicators, such as well-being and life satisfaction. Unlike traditional unidimensional approaches, dual-factor models highlight that the absence of mental illness does not necessarily imply the presence of well-being, and conversely, individuals may experience psychological distress while maintaining positive functioning (Magalhães, 2024) his framework thus provides a more comprehensive understanding of mental health, emphasizing the importance of both alleviating symptoms and fostering positive psychological resources.

Grounded in this multidimensional understanding of mental health, anxiety and depression are among the most common mental health symptoms, affecting millions of individuals and significantly impairing quality of life, relationships, and occupational functioning (Kessler et al., 2005; WHO, 2022). They frequently co-occur with somatization, which involves the manifestation of physical symptoms that medical conditions cannot fully explain (Mewes, 2022). Somatization is strongly associated with psychological distress, including elevated levels of anxiety and depression (Barsky et al., 2005; López-Santiago & Belloc, 2012). This mental health and well-being (de Vries et al., 2023). Life satisfaction, the cognitive and emotional assessment of one’s life, reflects personal achievements and the overall quality of lived experiences (Lucas et al., 1996). Research has consistently demonstrated a negative correlation between life satisfaction and symptoms of depression, anxiety, and other psychological disorders (López-Ruiz et al., 2021; Vera Villarroel et al., 2012). Moreover, life satisfaction is linked to other positive psychological outcomes, such as increased self-esteem and optimism, reinforcing its centrality in understanding mental health dynamics (Bhattacharyya et al., 2025; Szcześniak et al., 2021) interplay complicates diagnosis and treatment, highlighting the need for integrated intervention strategies. Recognizing these interconnected symptoms is essential for developing effective, person-centered mental health interventions.

Complementing this focus on clinical symptoms, life satisfaction emerges as a key component in understanding). Studies have shown that individuals with higher levels of life satisfaction are better equipped to cope with stress and less vulnerable to the adverse effects of psychological disorders (Boreham & Schutte, 2023). This protective role is closely tied to constructs such as purpose in life, which has been shown to buffer against the impact of stress and promote resilience (Arráez & Castedo, 2018; Creswell et al., 2005). Therefore, life satisfaction emerges as a key variable for holistically assessing the student’s health (Norambuena-Paredes et al., 2025).

However, it is essential to acknowledge that the relationship between psychological distress and well-being is complex. While life satisfaction contributes to positive outcomes, they do not eliminate the risk of anxiety or depression (Cornwell et al., 2024). Research highlights that a diminished sense of purpose, a key component of life satisfaction, heightens stress sensitivity, reflected in increased autonomic responses to emotional stressors (Ishida & Okada, 2006). Conversely, a strong sense of purpose is linked to greater behavioral activation and lower depressive and anxious symptoms, emphasizing its role in psychological resilience (Boreham & Schutte, 2023).

In light of the considerations stated in the preceding paragraph, the assessment of life satisfaction and symptomatology related to anxiety, depression, and somatization necessitates the use of reliable and valid measurement tools. Psychometric screening instruments are widely used in primary healthcare settings (Habtamu et al., 2022), where time and resources are often limited. Data from a systematic review emphasize the need for tools that balance simplicity with diagnostic accuracy, enabling healthcare providers to screen large populations effectively without requiring extensive training or diagnostic expertise (Ali et al., 2016). By integrating validated screening tools into routine care, health systems can identify individuals at risk for mental health disorders and provide timely interventions that address symptoms and underlying processes; early and systematic screening is essential for identifying issues and enabling timely intervention and support. While primary care has led the way in implementing brief and reliable mental health assessments, similar tools are increasingly relevant in other high-stress environments. University settings, in particular, benefit from scalable screening strategies that can be seamlessly integrated into existing support systems. Such services may involve assessment, psychotherapy, counseling, medication management, and referral to specialized or residential care when appropriate (Osborn et al., 2022). Therefore, incorporating assessments for depression, anxiety, somatization, and life satisfaction offers a more comprehensive understanding of students’ overall well-being, ensuring more targeted and effective care (Boone et al., 2022; Forbes et al., 2021).

In this respect, mental health screening in university students is increasingly important as rates of psychological distress, including depression, anxiety, and somatization, rise within this population (Gavurova et al., 2022). University life introduces academic, social, and financial pressures that significantly impact students’ well-being (Campbell et al., 2022). Measures such as the Generalized Anxiety Disorder—7 (GAD-7), Patient Health Questionnaire—9 (PHQ-9), Somatic Symptom Scale—8 (SSS-8), and Satisfaction with Life Scale (SWLS) have been used to screen university students for mental health concerns in various countries (Byrd-Bredbenner et al., 2020; Goweda et al., 2022; Rogowska et al., 2021; Rufino et al., 2024). The scientific literature supports evidence for substantial comorbidity between physical symptoms and anxiety and mood disorders in multiple populations (Fujii et al., 2018; Gavurova et al., 2022; Kong et al., 2022), thus highlighting the importance of using integrated screening tools—such as the GAD-7, PHQ-9, and SSS-8—to comprehensively assess the multifaceted nature of psychological distress.

Building on this argument, Ali et al. (2016) state that the PHQ-9, a screening tool for symptoms of depression, performed well in two large studies conducted on university students from Nigeria and China. Studies in Latin America have found it is a reliable instrument to use in this population (López-Guerra et al., 2022; Errazuriz et al., 2022; Campo-Arias et al., 2023; Saldivia et al., 2019). A two-dimensional factor structure was also observed for the PHQ-9 that adequately fits the data from Colombian university students in the health field (Campo-Arias et al., 2023). Similar results were found in a study involving Ecuadorian university students, demonstrating adequate psychometric properties in this population. The authors concluded that it is an easy-to-implement screening to design preventive programs for Ecuadorian university students at risk of developing depressive disorders (López-Guerra et al., 2022).

On the other hand, the GAD-7 is another frequently used screening tool in mental health settings in different cultures and countries. Validation of the GAD-7 has been performed on various university students’ samples. A study conducted among students in Bangladesh found Cronbach’s α coefficient to be 0.895, indicating high internal consistency (Dhira et al., 2021). Similar results are reported for university students in Latin America (Moreno-Montero et al., 2025, 2024; Villarreal-Zegarra et al., 2024). A study conducted in six LA countries, Bolivia, Colombia, Chile, Ecuador, El Salvador, and Paraguay, confirmed the unidimensional structure of the GAD-7, along with measurement equivalence across nationalities from the six countries. Additionally, it confirmed the validity and reliability of the GAD-7 in university students (Moreno-Montero et al., 2025).

Moreover, a scoping review and meta-analysis identified several measures used to screen somatic symptoms. The numerous studies reviewed demonstrate that the SSS-8 possesses reliable psychometric properties across various countries and populations (Hybelius et al., 2024). In a study involving Chinese college students, Wu et al. (2024) supported using the SSS-8 in assessing symptom burden within the university student population. Studies in Latin America have also validated the SSS-8. García et al. (2018) conducted descriptive and cross-sectional research on 401 participants from a clinical rural setting, with results suggesting the SSS-8 as a valuable tool with adequate psychometric properties: high internal consistency, good validity, and appropriate discriminative capacity. However, less is known about its psychometric functioning in Latin American university students.

On the other hand, the SWLS has shown adequate psychometric properties among university students worldwide as well, including Latin American countries such as México, Colombia, Brazil, Dominican Republic, and Ecuador (López-Guerra et al., 2022; Useche & Serge, 2017; Zerpa et al., 2023). Useche and Serge (2017) conducted a study in Colombia; their sample was composed of 150 university students with a mean age of 19.64, from several higher education institutions and with different academic degrees. The results prove that the scale has a good factorial solution and favorable internal consistency coefficients. Researchers found similar results among students in the Dominican Republic; the SWLS was proven as a valid, reliable, and applicable instrument to evaluate satisfaction with life in university students (Zerpa et al., 2023). The SWLS also emerges as a psychometrically adequate instrument in Mexican and Colombian university student samples (Norambuena-Paredes et al., 2025).

Furthermore, previous research has documented gender-based differences in mental health among university students (Castillo-Díaz et al., 2024; Erdemir & Kis, 2024; Nogueira et al., 2022). However, to ensure that any observed differences truly reflect variations in the latent construct rather than measurement artifacts, it is essential to establish measurement invariance across gender. Examining latent mean differences provides an opportunity to assess known-groups validity by testing whether the instruments discriminate between groups that are theoretically expected to differ (Steinmetz, 2018). These comparisons are only meaningful when measurement invariance is supported, ensuring that differences are attributable to the construct itself and not to biases or inconsistencies in the measurement model.

Drawing from the arguments presented above, the validation of psychometric tools to assess symptoms of depression, anxiety, somatization, and life satisfaction is of paramount importance. Reliable and culturally relevant instruments provide the foundation for effective mental health care, enabling the accurate identification of needs and the tailoring of interventions to specific populations, like university students. Screening tools for symptoms of depression and anxiety have been widely utilized in diverse settings, demonstrating their utility in identifying psychological distress (Ali et al., 2016). However, the validity and reliability of these instruments must be continuously assessed to ensure their applicability across cultural and contextual variations.

In this regard, understanding the specific cultural and contextual factors affecting university students in Honduras becomes essential for ensuring the relevance and accuracy of these tools. This is especially important because Honduras presents a unique sociocultural and structural context: mental health service provision is limited (McBoyle, 2021). The country also faces high poverty and inequality, and one of the highest Gini index scores in Latin America. While some of these challenges are shared with neighboring Central American Countries, Honduras has the lowest Human Development Index (HDI) score of the region (0.645 in 2023), reflecting the structural and social challenges in the country (United Nations, 2025). Moreover, the country also shows one of the largest socioeconomic gaps in higher education access in Central America (SEGIB, 2024). Exposure to violence and adverse childhood experiences is also high, which may shape how symptoms like depression, anxiety, and somatization are experienced and reported (Landa-Blanco et al., 2024b). These contextual factors suggest that instruments validated elsewhere may, hypothetically, behave differently in the Honduran student population, evidencing the need to assess their validity and reliability in this specific setting. Depression and anxiety are prevalent among Honduran University students (Castillo-Díaz et al., 2024; Landa-Blanco et al., 2022), often impairing students’ academic performance and daily functioning (Castillo-Díaz et al., 2022; Landa-Blanco et al., 2024a). Although the GAD-7 and the PHQ-9 have been previously used in the Honduran setting (Landa-Blanco et al., 2024a), the psychometric properties of both instruments have not been established beyond their internal consistency in this setting. To the best of our knowledge, no published research has specifically examined somatization as a distinct variable among Honduran university students. Considering these factors, the present study sought to evaluate the structural, convergent, and discriminant validity, measurement invariance, and internal consistency of the GAD-7, PHQ-9, SSS-8, and SWLS among Honduran university students. The validation of these instruments is justified given their extensive international use as brief and psychometrically sound measures of anxiety, depression, somatic distress, and subjective well-being. However, cultural variations in symptom presentation, linguistic interpretation, and response tendencies may influence their psychometric performance. Establishing their reliability and factorial structure in this context is essential to ensure accurate assessment and facilitate meaningful cross-cultural comparisons in mental health research.

## 2. Materials and Methods

### 2.1. Instruments

#### 2.1.1. Generalized Anxiety Disorder—7

The Generalized Anxiety Disorder—7 (GAD-7) measures symptoms of anxiety reported during the past two weeks before the evaluation. Individual items of the GAD-7 are rated on a frequency-based 4-point Likert-type scale, ranging from 0 (“not at all”) to 3 (“nearly every day”). Total scores are calculated summatively, ranging between 0 and 21; higher scores indicate a more frequent occurrence of anxiety symptoms. Data from the original validation of the GAD-7 suggest a unidimensional factor structure (Spitzer et al., 2006). The Spanish version of the GAD-7 was used in the present study, with authors reporting high internal consistency (*α* = 0.936) (García-Campayo et al., 2010).

#### 2.1.2. Patient Health Questionnaire—9

The Patient Health Questionnaire—9 (PHQ-9) measures symptoms of depression reported during the past two weeks before the evaluation. Individual items are rated on a frequency-based four-point Likert-type scale, ranging from 0 (“not at all”) to 3 (“nearly every day”). Total scores are calculated summatively, ranging between 0 and 27; higher scores indicate a more experienced frequency of depression symptoms. Data from the original validation of the PHQ-9 suggest a unidimensional factor structure and a high internal reliability (*α* = 0.89) (Kroenke & Spitzer, 2002). The Spanish-language version of the PHQ-9 was used in the current study (Campo-Arias et al., 2023).

#### 2.1.3. Somatic Symptom Scale—8

The Somatic Symptom Scale—8 (SSS-8) is an eight-item, five-point Likert-type questionnaire that assesses somatic burden reported during the past week before the evaluation. Each item is rated based on its perceived burden (0 = “not at all”; 4 = “very much”). Total scores are calculated summatively, ranging between 0 and 32; higher scores indicate higher symptomatology. Data from the original validation of the GAD-7 suggest a unidimensional factor structure (Gierk et al., 2014). The Spanish-language version of the SSS-8 was used in the current study, this version has shown adequate reliability for the Honduran general population (*Ω* = 0.91) (Landa-Blanco et al., 2024b).

#### 2.1.4. Satisfaction with Life Scale

The Satisfaction with Life Scale (SWLS) is a five-item, seven-point Likert-type questionnaire (1 = “strongly disagree”; 7 = “strongly agree”). Total scores are calculated summatively, ranging between 5 and 35; higher scores indicate more satisfaction with life. Data from the original validation of the SWLS suggest a unidimensional factor structure (Diener et al., 1985). The Spanish version of the SWLS was used in this study, for which the authors report high internal consistency (*α* = 0.84) (Garrido Muñoz de Arenillas et al., 2010).

### 2.2. Participants

The National Autonomous University of Honduras (UNAH), the largest tertiary education center in the country, had 44,227 registered undergraduate students in its central campus as of the first trimester of 2023. At a 99% confidence level and a margin of error of 5%, the minimum required sample size was set at 654 respondents. However, the current study managed to collect data from 910 students. Women accounted for 67.45% (*n* = 613) of the sample, while the remaining 32.6% (*n* = 297) of the participants identified as men. Ages varied between 18 and 66 years, averaging 24.00 (*SD* = 6.05).

Inclusion criteria included: (1) being 18 years or older, (2) being enrolled in the university during the first academic trimester of 2023, and (3) being enrolled in an undergraduate degree. The sample selection process followed a non-probabilistic strategy by volunteers. The online survey was disseminated in the following ways: (1) on-campus visits (to classrooms and social areas) and (2) institutional emails. Participation was voluntary; no academic, social, or financial incentives were given to the participants.

### 2.3. Data Analysis

To confirm the contextual adequacy of the instruments for the Honduran university population, we conducted a qualitative appraisal involving two complementary groups: a panel of psychology experts and a focus group of 10 students. The panel of experts, composed of six professionals with experience in clinical assessment and psychometrics, evaluated each item for conceptual relevance, cultural appropriateness, and potential sources of ambiguity. Concurrently, the student group reviewed the items to assess comprehensibility, clarity of language, and ease of response from the perspective of individuals directly affected by these measures. This two-tiered evaluation allowed for the identification of items that might require linguistic adaptation or contextual modification to ensure accurate measurement. The review process revealed that all items were both understandable and culturally appropriate, and no modifications to the adopted versions were necessary. This step thus provided additional confidence that the instruments could reliably capture depression, anxiety, somatization, and life satisfaction in Honduran university students.

During the data collection, all survey items were required to be answered, ensuring a complete dataset with no missing responses. Prior to analysis, normality of the distributions was assessed using skewness and kurtosis indices, as well as visual inspection of histograms and Q-Q plots. Skewness and kurtosis values ranging from −2 to +2 were deemed indicative of acceptable univariate normality (Mishra et al., 2019); all variables demonstrated acceptable normality, and no transformations were necessary. Consequently, no participants were excluded during the data cleaning process, and the dataset was deemed appropriate for the planned statistical procedures.

The initial phase of the analysis entailed reporting descriptive statistics for each item and total scores of the scales, including measures of central tendency (mean, standard deviation, and median), and distributional properties (minimum and maximum values, skewness, and kurtosis). Subsequently, a confirmatory factor analysis (CFA) for the full sample (single group) was performed to evaluate the structural validity of each instrument. All models were specified as one-factor structures based on prior evidence and theoretical expectations. No additional parameter restrictions or associated residuals were applied. The CFA utilized the weighted least squares mean and variance adjusted (WLSMV) estimate method for categorical data (Lomax, 2015). The model fit was assessed using the following indices: comparative fit index (CFI), normed fit index (NFI), Tucker–Lewis Index (TLI), and root mean square error of approximation (RMSEA). CFI, NFI, and TLI values of ≥0.90 indicate an acceptable model fit, whereas values of ≥0.95 suggest a good model fit (Hu & Bentler, 1999). Conversely, a RMSEA of ≤0.08 was an indicator of an acceptable model fit (Hu & Bentler, 1999). Statistically significant factor loadings of λ ≥ 0.40 (*p* < 0.05) were deemed acceptable for supporting the adequacy of the measurement models (Cheung et al., 2024). The McDonald Omega index (*Ω*) and the composite reliability (CR) index evaluated the internal consistency of the scales. As an indicator of convergent validity previously considered in the literature (Cheung et al., 2024), we report the average variance extracted (AVE). Values exceeding 0.50 are acceptable indicators of convergent validity, showing that the latent variable accounts for a minimum of 50% of the variance in its observable variables (Cheung et al., 2024).

A multigroup confirmatory factor analysis (MGCFA) was used to evaluate measurement invariance between women and men. Three models of invariance were examined. The initial model (configural invariance) assessed whether the unidimensional structure of the scales was consistent across groups. The second model (metric invariance) examined the equivalence of factor loadings across groups. The third model (scalar invariance) assessed the equivalence of response thresholds for latent traits (Gomes et al., 2022). When full invariance was not achieved at a given level (metric or scalar), partial invariance was tested by gradually freeing one parameter at a time (i.e., factor loadings or thresholds) based on modification indices until acceptable model fit was reached (Gomes et al., 2022).

This study evaluated the configural model using the same criteria as the CFA for the full sample analysis. The metric and scalar models were assessed according to literature recommendations, with ΔCFI < 0.010 and ΔRMSEA ≤ 0.015, compared to the configural model, deemed indicative of model invariance (Chen, 2007). Once scalar or partial scalar invariance was established, latent mean comparisons were conducted to examine known-groups validity. Latent mean comparisons allow the analysis of group differences in the underlying construct free from measurement error, providing a more precise estimate than observed scores (Steinmetz, 2018). The group “women” was set as the reference (mean fixed to zero), and latent means for “men” were freely estimated. Significant latent mean differences were interpreted as evidence that the instruments discriminate between groups as theoretically expected. In addition to statistical significance (*z*-tests), effect sizes (Cohen’s *d*, obtained from the standardized latent mean differences) were reported to quantify the magnitude of group differences, with values of 0.20, 0.50, and 0.80 typically interpreted as small, medium, and large, respectively (López-Martín & Ardura, 2023).

Finally, to assess the validity based on its relationship with other constructs (convergent and discriminant validity), a Pearson correlation analysis was conducted utilizing the factor scores obtained from the one-dimensional analysis performed during the structural validity phase. Considering the reported comorbidity among symptoms of anxiety, depression, and somatization (Kong et al., 2022; Tu et al., 2024; Velasco-Durantez et al., 2024), convergent validity was assessed through the correlation between the GAD-7, PHQ-9, and SSS-8. Divergent validity was examined by analyzing the inverse correlation between life satisfaction (SWLS) with the GAD-7, PHQ-9, and SSS-8. The correlation coefficients were interpreted following criteria previously established in the literature (Swank & Mullen, 2017). Statistical significance was determined at the threshold of *p* < 0.05. All analyses were conducted using R software (version 4.4.2) through the packages semTools version 0.5–6 (Jorgensen et al., 2022), and lavaan version 0.6–16 (Rosseel et al., 2024).

### 2.4. Ethical Considerations

The study was approved by the Research Ethics Committee of the Faculty of Social Sciences of the National Autonomous and registered under protocol CEIFCS-2023-P2. All subjects were required to give informed consent, and data was collected anonymously.

## 3. Results

Table 1 presents the descriptive statistics for both item-level and total scores of each scale. Overall, individual item scores tend to cluster around central values. All skewness and kurtosis values fall between −2 and +2, indicating acceptable univariate normality across items.

Table 2 presents the fit indices obtained for the GAD-7, PHQ-9, SSS-8, and SWLS models. All models depict adequate fit indices with CFI, NFI, and TLI ≥ 0.95 and RMSEA < 0.08, corroborating the unidimensional factorial structure of the measures.

Factor loadings and reliability indices of the scales are presented in Table 3. GAD-7 demonstrated factor loadings ranging from 0.780 (GAD 6) to 0.926 (GAD 3), all of which were significant (*p* < 0.001), confirming that each item is a robust indicator of the latent factor of generalized anxiety. Internal consistency was high (*Ω* = 0.927; CR = 0.948). Additionally, the AVE of 0.724 showed that the factor explains more than 72% of the variance in the items.

PHQ-9 showed factor loadings between 0.741 (PHQ 9) and 0.866 (PHQ 2), with all items surpassing the threshold of 0.70. Internal consistency was excellent (*Ω* = 0.919; CR = 0.940). Furthermore, the AVE of 0.635 suggested that the items explain a significant proportion of the variance in the depression construct.

For SSS-8, factor loadings ranged from 0.615 (SSS 1) to 0.787 (SSS 5). Internal consistency was adequate (*Ω* = 0.873; CR = 0.897). The AVE of 0.524, although slightly above the threshold of 0.50, indicates that the factor accounts for a sufficient proportion of the variance in the items.

The SWLS displayed factor loadings between 0.671 (SWLS 5) and 0.907 (SWLS 3), with most items exceeding 0.70, except for SWLS 5, which, while slightly lower, is still deemed acceptable. Internal consistency was good, with a McDonald’s Omega of 0.875, and CR reached 0.895. The AVE of 0.632 reflects the construct’s strong ability to explain the item variance.

The results of the measurement invariance are shown in Table 4. The findings indicate configural, metric, and scalar invariance for GAD-7, SSS-8, and SWLS. For the PHQ-9, configural and scalar invariance were achieved. Although the metric model met the ΔCFI criterion (0.004), it did not meet the ΔRMSEA criterion (0.025). Guided by the modification indices, the loading of Item 4 was freed, resulting in partial metric invariance with acceptable fit indices (ΔCFI < 0.01; ΔRMSEA ≤ 0.015).

Latent mean comparisons present statistically significant differences between women (reference group) and men across all four instruments. Women showed higher latent means for anxiety (GAD-7; latent mean difference [ΔM] = −0.43; standard error [*SE*] = 0.06; *z* = −6.77; *p* < 0.001; *d* = −0.52), depression (PHQ-9; ΔM = −0.30; *SE* = 0.06; *z* = −4.90; *p* < 0.001; *d* = −0.37), and somatization (SSS-8; ΔM = −0.39; *SE* = 0.05; *z* = −7.14; *p* < 0.001; *d* = −0.58), indicating greater symptom severity among women. In contrast, men showed higher levels of life satisfaction (SWLS; ΔM = 0.19; *SE* = 0.06; *z* = 3.05; *p* = 0.002; *d* = 0.23). Effect sizes ranged from small to moderate, with the largest difference observed for somatization.

As an indicator of convergent validity, Table 5 depicts the correlations between the factor scores of the scales. The findings indicate statistically significant correlations between the four scales (*p* < 0.001). The PHQ-9, GAD-7, and SSS-8 show large positive correlations (*r* > 0.600), reflecting a tendency towards possible high comorbidity between the constructs evaluated. In contrast, the SWLS demonstrated significant moderate inverse associations with the GAD-7, PHQ-9, and SSS-8, with correlation coefficients ranging from −0.315 to −0.475.

## 4. Discussion

This study sought to investigate the psychometric properties of screening measures for depression, anxiety, somatization, and life satisfaction in Honduran university students. The results showed structural, convergent, and discriminant validity, measurement invariance for gender, and adequate indicators of reliability indices for each of the scales analyzed, supporting the usefulness of these tools in detecting mental health problems and assessing the subjective well-being of university students in Honduras.

The structural fit indices of each scale meet and exceed established thresholds, demonstrating that these scales are psychometrically robust within the Honduran context. This confirms their cross-cultural applicability, addressing a critical gap in screening tools for mental health assessment in Honduras. Furthermore, the scales exhibited an adequate fit, aligning with previous findings (López-Ortega et al., 2016; Pollo et al., 2022; Rosario-Hernández et al., 2023; Villarreal-Zegarra et al., 2024). The CFI, TLI, and NFI values were consistently high across the four tested unidimensional models (≥0.995). Psychometric literature supports the plausibility of such elevated fit indices in well-identified unidimensional models characterized by strong factor loadings and low residual variance (Brown, 2015; Lomax, 2015).

The GAD-7 and PHQ-9 scales effectively measure symptoms of anxiety and depression, as their items capture the core manifestations of these constructs. Similarly, the SWLS and SSS-8 scales reliably assess life satisfaction and somatic symptoms. However, contextualizing assessments through interviews remains essential for a more personalized and effective intervention. Understanding the factors that influence health and patients’ experiences enhances the development of tailored interventions for stress, anxiety, somatization, and overall patient well-being (Cahill et al., 2024; Ciarrochi et al., 2024).

Likewise, the factor loadings for the PHQ-9, GAD-7, and SWLS-3 scales were high, resulting in a unidimensional structure for these instruments, which aligns with previous studies (López-Ortega et al., 2016; Pollo et al., 2022; Rosario-Hernández et al., 2023; Villarreal-Zegarra et al., 2024). On the other hand, the SSS-8 scale demonstrated acceptable factor loadings, as its items exceeded the established benchmark. However, items such as SSS-1 (“Headaches”) exhibited a moderate loading, reflecting sociocultural differences in the perception of somatic symptoms (Löwe & Gerloff, 2018). Despite this, the AVE of the SSS-8 exceeded the minimum threshold, confirming its convergent validity. Regarding internal consistency, all scales demonstrated excellent reliability, aligning with international standards (Hybelius et al., 2024; López-Ortega et al., 2016; Lutkiewicz et al., 2024; Pollo et al., 2022; Rosario-Hernández et al., 2023; Sameie-Sis et al., 2024; Villarreal-Zegarra et al., 2024), highlighting the importance and applicability of these screening instruments in clinical settings in Honduras.

The PHQ-9, GAD-7, SSS-8, and SWLS are robust psychometric tools for use in Honduras. Their invariance supports their reliable use for comparing anxiety, somatization, and life satisfaction across genders. The SWLS demonstrated perfect invariance, highlighting it as an optimal tool for assessing subjective well-being in multicultural contexts (Swami et al., 2025). For the PHQ-9, configural, partial metric (after freeing Item 4: “low energy/fatigue” based on modification indices), and scalar invariance were achieved. This finding allows for the comparison of latent means across gender while acknowledging minor measurement non-equivalence, which should be considered when interpreting gender differences in depression. This aligns with previous studies, where the expression of internalizing symptoms varies according to gender-cultural norms, and the observed differences may obscure cultural biases (Krishnan & Ashokkumar, 2023).

The latent mean comparisons revealed statistically significant gender differences across all scales, with effect sizes ranging from small to moderate. These findings are consistent with previous research indicating that women tend to report lowest scores in mental health outcomes than men (Castillo-Díaz et al., 2024; Landa-Blanco et al., 2021; Nogueira et al., 2022). Nevertheless, these results should be interpreted with some caution, particularly for depression, as minor non-equivalence in the PHQ-9 may influence the magnitude of the observed differences. Overall, these findings provide preliminary support for known-groups validity.

This study reveals patterns of positive and statistically significant correlations between the GAD-7 (anxiety), PHQ-9 (depression), and SSS-8 (somatization), a relationship that has been reported in previous literature based on Latin American university students (Landa-Blanco et al., 2021). The strongest correlation between the GAD-7 and PHQ-9 reflects symptomatic convergence, such as symptoms like fatigue, irritability, and sleep disturbances, which are shared diagnostic criteria for both conditions (An et al., 2019; He et al., 2023; Pizzagalli, 2016). This underscores the need for integrated approaches in clinical practice, where the assessment and management of comorbid conditions should be incorporated. The negative correlations between the SWLS and the other scales suggest that subjective well-being is a buffer against adverse symptomatology. Positive emotions expand the behavioral and cognitive repertoire in this context, facilitating resilience (Burgdorf et al., 2017).

The findings of this study hold important implications for mental health screening among university students, particularly in low-resource settings like Honduras. The demonstrated psychometric robustness of the PHQ-9, GAD-7, SSS-8, and SWLS supports their use as efficient and culturally appropriate tools for identifying students at risk of depression, anxiety, somatization, and low subjective well-being. Given the high comorbidity between these conditions (Meule & Voderholzer, 2020), early and accurate detection is essential for guiding timely interventions and preventing long-term psychological impairment in university students (Emmerton et al., 2024). The ability to capture these constructs through brief self-report measures allows institutions to implement large-scale screening initiatives, which are especially critical in university environments where mental health problems are prevalent yet often underdiagnosed (Mugotitsa et al., 2025). Moreover, the inverse relationship observed between life satisfaction and symptom severity highlights the value of incorporating well-being assessments into routine mental health evaluations. Promoting positive mental health, not merely the absence of distress, should be a central component of prevention strategies. These findings provide a foundation for evidence-based mental health monitoring and inform institutional policies aimed at supporting student well-being through targeted programs, referrals, and psychoeducational interventions.

In this sense, demonstrating that the PHQ-9, GAD-7, SSS-8 and SWLS are valid and reliable among Honduran university students of the UNAH provides the Vice-Rectorate for Student Affairs and Guidance (VOAE UNAH) with empirically grounded tools to assess and monitor student mental health and well-being. As VOAE offers a comprehensive range of health services including general medicine, dentistry, psychology, nutrition, physical therapy, clinical laboratory, pharmacy, nursing, and a women’s health program (UNAH, 2025), these validated instruments can be integrated across its areas to identify emotional and psychosomatic needs that affect overall health. Their application supports coordinated, evidence-based care that enhances prevention, treatment, and referral processes, strengthening VOAE’s capacity to promote holistic student well-being.

Despite the study’s practical and contextual relevance, certain limitations must be acknowledged. First, quantitative research on university mental health with a non-probabilistic sample presents significant constraints. The generalizability of findings is limited, as the sample does not adequately represent the broader student population. Selection bias is also a concern, given that not all students had an equal probability of participation. Furthermore, the sample may lack representativeness regarding age, gender, and socioeconomic diversity. These limitations not only restrict the external validity of the findings but also raise concerns about whether the observed patterns truly reflect the broader university context or are artifacts of the recruitment process. Second, the study did not include a test–retest reliability assessment, which limits conclusions about the temporal stability of the measures (Park et al., 2018). Third, although gender-based known-groups analyses provided preliminary evidence of discriminative validity, further contrasts with more distinct groups (e.g., clinical vs. non-clinical) are needed to strengthen the conclusions. Fourth, the assessment of convergent and discriminant validity was also indirect, given that no alternative measures of the same constructs were employed, and negative correlations with life satisfaction provide only a limited test of discriminant validity. Collectively, these limitations should be carefully considered when interpreting the results.

In this study, the psychometric analysis of the PHQ-9, SSS-8, GAD-7, and SWLS was conducted from a latent variable perspective, allowing for the assessment of the underlying factorial structure of the psychological constructs examined. However, subsequent investigations could incorporate alternative psychometric approaches, such as network analysis (Qian et al., 2025), to explore the interconnections between symptoms and the relationships among different nodes. This alternative framework would enable the identification of central and key symptoms in the connection between depression, anxiety, and somatization, along with indicators of life satisfaction, providing a more dynamic understanding of their interrelations.

Future research could adopt a longitudinal approach to track anxiety, depression, and somatization symptoms among Honduran university students, providing insight into their long-term impact on life satisfaction. Qualitative studies could further explore students’ lived experiences and coping mechanisms (Allen et al., 2024), while comparative analyses could assess symptom prevalence across demographic subgroups. Additionally, intervention studies could evaluate the efficacy of various strategies (Amaro et al., 2025), ranging from individualized therapies to institution-wide mental health initiatives, in symptom reduction and overall well-being improvement. Finally, future research should explore external and diagnostic variables to establish more precise threshold scores for the GAD-7, PHQ-9, SSS-8, and SWLS in Honduran university students. This would enhance the clinical utility of these scales by improving their sensitivity and specificity in identifying relevant psychological conditions.

## 5. Conclusions

This study confirms that the GAD-7, PHQ-9, SSS-8, and SWLS are psychometrically robust tools for assessment within the Honduran university context, highlighting their cross-cultural applicability and clinical utility. Their use extends to training healthcare professionals in screening tests, ensuring contextually adapted implementation for the early detection of mental health issues. This, in turn, facilitates the development of comprehensive intervention strategies incorporating multidimensional assessment protocols and treatments that prioritize overlapping symptoms. These findings contribute to evaluating mental health in Honduras by generating evidence for a holistic and culturally sensitive approach. They underscore the critical need for reliable and valid instruments to design appropriate intervention strategies.

## Figures and Tables

**Table 1:** Descriptive statistics of the GAD-7, PHQ-9, SSS-8, and SWLS items.

Model	Item	*M*	*SD*	Mdn	Min	Max	Skewness	Kurtosis
GAD-7	GAD 1	1.67	1.01	2	0	3	0.01	−1.19
	GAD 2	1.58	1.03	1	0	3	0.03	−1.17
	GAD 3	1.81	1.02	2	0	3	−0.26	−1.13
	GAD 4	1.73	1.04	2	0	3	−0.18	−1.19
	GAD 5	1.49	1.07	1	0	3	0.07	−1.25
	GAD 6	1.67	1.03	2	0	3	−0.07	−1.19
	GAD 7	1.49	1.13	1	0	3	0.06	−1.39
	Total GAD-7	11.43	6.08	11	0	21	−0.03	−1.03
PHQ-9	PHQ 1	1.39	1.01	1	0	3	0.22	−1.04
	PHQ 2	1.48	1.05	1	0	3	0.11	−1.20
	PHQ 3	1.56	1.13	2	0	3	−0.05	−1.38
	PHQ 4	1.88	0.97	2	0	3	−0.30	−1.05
	PHQ 5	1.50	1.10	1	0	3	0.03	−1.31
	PHQ 6	1.38	1.13	1	0	3	0.18	−1.36
	PHQ 7	1.45	1.08	1	0	3	0.09	−1.25
	PHQ 8	1.13	1.06	1	0	3	0.46	−1.04
	PHQ 9	0.86	1.09	0	0	3	0.89	−0.67
	Total PHQ-9	12.62	7.37	12	0	27	0.15	−0.90
SSS-8	SSS 1	1.34	1.28	1	0	4	0.53	−0.87
	SSS 2	1.92	1.34	2	0	4	0.01	−1.18
	SSS 3	1.43	1.29	1	0	4	0.50	−0.87
	SSS 4	1.86	1.37	2	0	4	0.07	−1.21
	SSS 5	1.26	1.38	1	0	4	0.66	−0.94
	SSS 6	1.11	1.29	1	0	4	0.84	−0.52
	SSS 7	2.28	1.38	2	0	4	−0.19	−1.26
	SSS 8	1.89	1.50	2	0	4	0.09	−1.40
	Total SSS-8	13.10	7.79	13	0	32	0.21	−0.79
SWLS	SWLS 1	4.24	1.50	4	1	7	−0.21	−0.02
	SWLS 2	4.77	1.44	5	1	7	−0.43	0.01
	SWLS 3	4.66	1.68	5	1	7	−0.42	−0.46
	SWLS 4	4.60	1.63	5	1	7	−0.34	−0.52
	SWLS 5	3.95	1.92	4	1	7	0.02	−1.03
	Total SWLS	22.22	6.66	22	5	35	−0.19	−0.32

Note. *M* = Mean; *SD* = Standard deviation; Mdn = Median; Min = Minimum; Max = Maximum.

**Table 2:** Fit indices of the tested models.

Model	*χ*^2^ (df)	*p*	CFI	NFI	TLI	RMSEA (CI 90%)
GAD-7	62.180 (14)	<0.001	0.999	0.998	0.998	0.062 (0.046, 0.078)
PHQ-9	118.767 (27)	<0.001	0.997	0.996	0.996	0.061 (0.050, 0.073)
SSS-8	59.911 (20)	<0.001	0.996	0.995	0.995	0.047 (0.033, 0.061)
SWLS	6.810 (5)	<0.001	0.999	0.999	0.999	0.020 (0.001, 0.053)

Note. *χ*^2^ (df) = chi-square and degrees of freedom; *p* = *p*-value; CFI = Comparative fit index; NFI = Normed fit index; TLI = Tucker–Lewis Index; RMSEA = Root Mean Square Error of Approximation; CI = Confidence Interval.

**Table 3:** Factor loadings and reliability indices of the different scales.

Model	Item	λ	*Ω*	AVE	CR
GAD-7	GAD 1	0.865 *	0.927	0.724	0.948
	GAD 2	0.856 *			
	GAD 3	0.926 *			
	GAD 4	0.875 *			
	GAD 5	0.859 *			
	GAD 6	0.780 *			
	GAD 7	0.784 *			
PHQ-9	PHQ 1	0.765 *	0.919	0.635	0.940
	PHQ 2	0.866 *			
	PHQ 3	0.789 *			
	PHQ 4	0.787 *			
	PHQ 5	0.777 *			
	PHQ 6	0.843 *			
	PHQ 7	0.825 *			
	PHQ 8	0.770 *			
	PHQ 9	0.741 *			
SSS-8	SSS 1	0.615 *	0.873	0.524	0.897
	SSS 2	0.711 *			
	SSS 3	0.740 *			
	SSS 4	0.741 *			
	SSS 5	0.787 *			
	SSS 6	0.755 *			
	SSS 7	0.761 *			
	SSS 8	0.665 *			
SWLS	SWLS 1	0.782 *	0.875	0.632	0.895
	SWLS 2	0.791 *			
	SWLS 3	0.907 *			
	SWLS 4	0.808 *			
	SWLS 5	0.671 *			

Note. * *p* < 0.001; λ = Factor loadings, *Ω* = McDonald’s omega; AVE: Average variance extracted; CR: composite reliability.

**Table 4:** Configural, metric, scalar, and residual invariance of GAD-7, PHQ-9, SSS-8, and SWLS by gender.

Invariance Model	*χ*^2^ (df)	Δ*χ*^2^ (Δdf)	CFI	ΔCFI	RMSEA (90% CI)	ΔRMSEA
GAD-7						
Configural	72.932 (28)	-	0.999	-	0.059 (0.043, 0.076)	-
Metric	95.256 (34)	22.324 (6)	0.998	**0.001**	0.063 (0.048, 0.078)	**0.004**
Scalar	94.373 (47)	21.441 (19)	0.999	**0.000**	0.047 (0.033, 0.061)	**0.012**
PHQ-9						
Configural	124.512 (54)	-	0.998	-	0.054 (0.041, 0.066)	-
Metric	239.844 (62)	115.32 (8)	0.994	**0.004**	0.079 (0.069, 0.090)	0.025
Metric (P)	186.426 (61)	91.914 (7)	0.996	**0.002**	0.067 (0.056, 0.078)	**0.013**
Scalar	199.686 (79)	75.174 (25)	0.996	**0.002**	0.058 (0.048, 0.068)	**0.004**
SSS-8						
Configural	85.988 (40)	-	0.995	**-**	0.050 (0.036, 0.065)	**-**
Metric	98.143 (47)	12.155 (7)	0.995	**0.000**	0.049 (0.035, 0.063)	**0.001**
Scalar	130.099 (70)	44.111 (30)	0.994	**0.001**	0.043 (0.032, 0.055)	**0.007**
SWLS						
Configural	11.484 (10)	-	10.000	**-**	0.018 (0.000, 0.056)	**-**
Metric	12.221 (14)	0.737 (4)	10.000	**0.000**	0.012 (0.000, 0.040)	**0.006**
Scalar	24.264 (38)	12.780 (28)	10.000	**0.000**	0.012 (0.000, 0.000)	**0.006**

Note. Metric (P) = Partial metric invariance, obtained after freeing the loading of Item 4 of the PHQ-9; *χ*^2^ (df) = chi-square and degrees of freedom; Δ*χ*^2^ (Δdf) = change in chi-square and degrees of freedom; CFI = Comparative fit index; ΔCFI = change in CFI; RMSEA = Root mean square error of approximation; CI = Confidence interval; ΔRMSEA = change in RMSEA. Bold values indicate support for invariance (ΔCFI ≤ 0.010; ΔRMSEA ≤ 0.015).

**Table 5:** Correlations between GAD-7, PHQ-9, SSS-8, and SWLS.

Scale	GAD-7	PHQ-9	SSS-8	SWLS
GAD-7	-			
PHQ-9	0.765 *	-		
SSS-8	0.715 *	0.669 *	-	
SWLS	−0.475 *	−0.356 *	−0.315 *	-

Note. * *p* < 0.05.

## Data Availability

Data is contained within the article or Supplementary Material. The original contributions presented in this study are included in the article/Supplementary Material. Further inquiries can be directed to the corresponding author.

## Supplementary Materials

The following supporting information can be downloaded at: https://www.mdpi.com/article/10.3390/bs15111546/s1.
